# Cine MRI in the Evaluation of Pulmonary Arteriovenous Malformations

**DOI:** 10.7759/cureus.43148

**Published:** 2023-08-08

**Authors:** Savas Ozdemir, Sherif Elsherif, Minh Nguyen, Gregory Wynn

**Affiliations:** 1 Department of Radiology, University of Florida College of Medicine – Jacksonville, Jacksonville, USA

**Keywords:** hereditary hemorrhagic telangiectasia, contrast-enhanced computed tomography pulmonary angiography, cine magnetic resonance imaging, steady state free precession, pulmonary arteriovenous malformation

## Abstract

We present findings on cine magnetic resonance imaging (MRI) using steady-state free precession (SSFP) pulse sequences in a patient with pulmonary arteriovenous malformation (PAVM). The technique has the advantage of demonstrating the pulsation of lesions during the cardiac cycle on cine images. It may not replace but may complement other MRI sequences in the characterization of pulmonary lesions in selected cases. To our knowledge, no prior video of cine images of PAVM has been provided in the literature.

## Introduction

Pulmonary arteriovenous malformation (PAVM) is an abnormal communication between pulmonary arteries and pulmonary veins, creating a right-to-left shunt. PAVMs are usually associated with hereditary hemorrhagic telangiectasia (HHT), inherited in an autosomal dominant pattern There is an estimated cerebral abscess risk of 5%-19% and a 9%-18% risk of ischemic stroke in those who manifest PAVM [1].

Transthoracic contrast echocardiography (TTCE) is the preferred initial screening test. This test allows for potential shunt identification in suspected PAVM cases. TTCE has a high sensitivity and negative predictive value of 97% and 99%, respectively. Unfortunately, it has a high false positive rate and cannot localize lesions [2]. Contrast-enhanced computed tomography pulmonary angiography (CTPA) is the gold standard for the diagnosis, localization, and characterization of a PAVM in patients with positive TTCE [3]. Contrast-enhanced magnetic resonance angiography (MRA) has gained popularity as a diagnostic tool. Recently, non-contrast ultra-short echo time magnetic resonance imaging (UTE MRI) has been reported as an alternative to obviate potential gadolinium-based contrast media-related adverse effects [4]. The use of cine MRI sequences has only been rarely reported in the literature [5,6]. To our knowledge, no prior video of cine images of PAVM has been provided in the literature. This technique may complement currently used MRI sequences by demonstrating pulsation of the lesions on cine images.

## Case presentation

**Figure 1:**
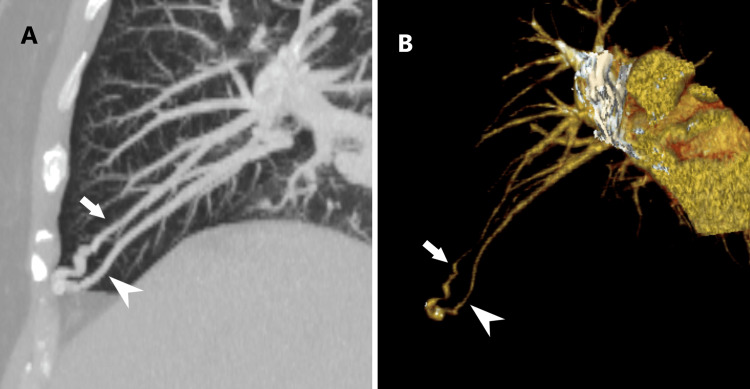
PAVM on CTPA (A) Sagittal oblique multiplanar reformatted maximum intensity projection image and (B) volume-rendered image from contrast-enhanced CT pulmonary angiography in a 53-year-old-woman shows the feeding artery (white arrow) and draining vein (white arrowhead) of a simple type pulmonary arteriovenous malformation, supplied by a pulmonary artery branch and draining into a pulmonary vein branch, respectively.

**Figure 2:**
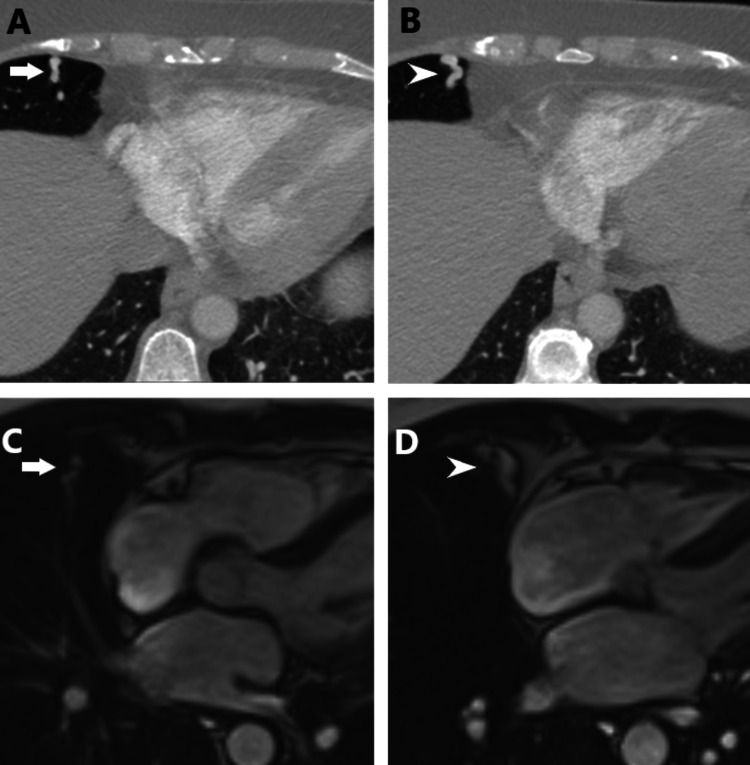
PAVM on CTPA and MRI Axial contrast-enhanced pulmonary angiography images (A, B) show the feeding artery site (white arrow in A) and draining vein site (white arrowhead in B) of a simple type of pulmonary arteriovenous malformation within the right middle lobe in a 53-year-old-woman. Corresponding steady-state free precession cine MRI images (C, D) show the feeding artery site (white arrow in C) and draining vein site (white arrowhead in D).

A 53-year-old female with a history of acute ischemic stroke had an extensive workup, including transesophageal echocardiogram (TEE), which suggested a patent foramen ovale (PFO). However, there was no PFO demonstrated on intracardiac echocardiography (ICE) imaging during attempted closure. A bubble study performed during ICE showed a late crossing of bubbles suggesting a pulmonary shunt. CTPA demonstrated a simple PAVM in the right middle lobe (Figures 1A, 1B, 2A-2D).

Incidentally, this PAVM appeared as a pulsating lesion on cine cardiac MRI images using steady-state free precession (SSFP) sequences (Video 1).

**Video 1:** PAVM on Cine MRI Steady-state free precession four chamber cine MRI images show pulsating vascular lesion in the right middle lobe in a 53-year-old-woman, consistent with pulmonary arteriovenous malformation.

## Discussion

Classification of PAVMs is based on the number of feeding arteries. The simple type has one segmental feeding artery, whereas the complex type has two or more segmental feeding arteries [7]. This classification does not use the number of sub-segmental arteries and draining veins. Endovascular embolization is indicated in cases where a feeding artery with a diameter of 2-3 mm or larger, measurably increasing size of PAVM, paradoxical emboli, or symptomatic hypoxemia, are present [8,9].

CT techniques, without and with contrast, are most commonly used in the initial evaluation and follow-up of patients with PAVM or risk of developing PAVM. Cumulative radiation dose may be substantial in patients with HHT secondary to repeated diagnostic and therapeutic interventions [10].

SSFP cine MRI sequence may not replace currently used MRI sequences since this sequence type and protocol is typically used for a small field of view rather than the entire lungs. It may complement currently used MRI sequences by demonstrating pulsation of the lesions on cine images.

The unique feature of the technique may help the characterization of pulmonary lesions since certain vascular and non-vascular abnormalities may mimic PAVM [11]. Non-vascular lesions, such as bronchoceles and tumors, may not show pulsation on cine MRI sequences.

## Conclusions

SSFP cine MRI demonstrates potential in aiding initial diagnosis and follow-up of PAVM in conjunction with other MRI sequences. Optimization of the technique and comparative studies are required to determine the value of this technique to complement established MRI sequences in selected cases.
